# Cognitive-behavioral treatment with behavioral activation for smokers with depressive symptomatology: study protocol of a randomized controlled trial

**DOI:** 10.1186/s12888-017-1301-7

**Published:** 2017-04-08

**Authors:** Elisardo Becoña, Carmela Martínez-Vispo, Carmen Senra, Ana López-Durán, Rubén Rodríguez-Cano, Elena Fernández del Río

**Affiliations:** 1 Smoking Cessation and Addictive Disorders Unit, Department of Clinical Psychology and Psychobiology, Faculty of Psychology, University of Santiago de Compostela, 15782 Santiago de Compostela, Galicia Spain; 2 Department of Psychology and Sociology, University of Zaragoza, 50009 Zaragoza, Spain

**Keywords:** Smoking cessation, Depressive symptoms, Behavioral activation, Randomized controlled trial

## Abstract

**Background:**

Smoking is an important risk factor for mental health-related problems. Numerous studies have supported a bi-directional association between cigarette smoking and depression. Despite the advances in understanding the comorbidity between both problems, the most effective psychological treatment that simultaneously targets smoking and depressive symptomatology remains unclear. The objective of this study is to assess the effectiveness of a cognitive-behavioral intervention for smoking cessation with components of behavioral activation for managing depressed mood.

**Method:**

A single blind, three-arm, superiority randomized controlled trial is proposed. Participants will be smokers over 18 years old, who smoke at least 8 cigarettes per day. Participants will be randomized to one of three conditions, using a 2:2:1 allocation ratio: 1) standard cognitive-behavioral smoking cessation treatment; 2) standard cognitive-behavioral smoking cessation treatment plus behavioral activation; or 3) a three-month delayed treatment control group. The primary outcome measures will be biochemically verified point-prevalence abstinence (carbon monoxide in expired air) and significant change from baseline in depressive symptoms to the end of treatment, and at the 3-, 6-, and 12-month follow-up.

**Discussion:**

This study aims to assess the efficacy of a cognitive-behavioral intervention with behavioral activation components for smoking cessation and depressive symptoms, compared to a standard cognitive-behavioral intervention to quit smoking. As the relation between depressive symptoms, even at subclinical levels, and quitting smoking difficulties is well known, we expect that such intervention will allow obtaining higher abstinence rates, lower relapse rates, and mood improvement.

**Trial registration:**

ClinicalTrials.gov: NCT02844595. Retrospectively registered 19th July, 2016. The study started in January 2016, and the recruitment is ongoing.

## Background

Both smoking and depression are leading causes of disability, mortality, and morbidity worldwide [1–3]. The frequent co-occurrence of depression and smoking is well documented, and empirical work suggests that smoking-depression relations could be bi-directional [4, 5]. Epidemiological research have shown that mood disorders are more prevalent among smokers compared to nonsmokers from the general population [6–8], and it has been found that smoking is also associated with a greater risk of onset and persistence of depression [9]. In addition, among treatment-seeking smokers, several studies have found greater levels of mood disorders, in both lifetime and past-year diagnoses [10].

Evidence suggests that smokers from the general population with depressive symptoms or with a depressive disorder, compared to those without one, present higher levels of cigarette consumption, greater nicotine dependence, are more likely to suffer intense nicotine withdrawal symptoms and to fail when trying to quit smoking, have more difficulties to maintain abstinence, and have a higher risk of smoking relapse over time [5, 11–13]. Additionally, it has been shown that even very low levels of pretreatment depressive symptoms can impact smoking cessation treatment outcomes [14].

At the same time, studies conducted both in general and clinical populations have found that smoking cessation is related to mood improvement in people who are able to remain abstinent [15–17]. In a recent meta-analysis that included 26 studies assessing mental health in persons with and without psychiatric disorders, Taylor and colleagues [18] found that quitting smoking is associated with a decrease in depressive and anxiety symptoms, with a reduction in stress levels, and an improvement in quality of life.

The U.S. Preventive Services Task Force (USPSTF) found strong evidence that behavioral interventions substantially improve achievement of tobacco cessation [19]. Despite this, a reduction over time in abstinence rates has been shown following psychological and/or pharmacological smoking cessation treatment [20–22]. As recent studies suggest, this trend could be due to a change in treatment-seeking smokers, who show significantly greater self-reported depressive symptoms when compared to prior studies [20, 23].

In relation to the efficacy of smoking cessation treatments aimed at smokers with depression, Gierisch and colleagues [24] conducted a systematic review and meta-analysis of studies examining smoking cessation interventions for smokers with past or current depression. Their results showed a positive effect of adding a behavioral mood management component to smoking cessation intervention. Recently, van der Meer and colleagues [25] reviewed 33 trials that included smoking cessation interventions with specific mood management components. Their results, in line with previous ones, showed a significant positive effect of adding psychosocial mood management components to a standard smoking cessation intervention in smokers with current or past depression.

Behavioral activation (BA) may be considered as a parsimonious treatment option for depression, because it is less complex but just as effective as cognitive-behavioral therapy and anti-depressant medication [26, 27]. Moreover, Richards and colleagues [28], who conducted the largest trial of BA for depression to date, found that BA therapy is more time-efficient and cost-effective when compared to cognitive-behavioral treatment for depression, suggesting that BA should be a front-line treatment for depression. It consists of a structured intervention that uses the principles of operant conditioning to activate clients to increase rewarding experiences in their lives, the enjoyment of daily activities, and to reconnect with environmental positive reinforcement [29].

The central role of reinforcement in the BA approach makes this intervention a good option to be included in an intervention to quit smoking. Audrain-McGovern and colleagues [30] found that when depression-prone smokers quit smoking, they experience the loss of smoking reinforcement, a diminution in positive mood, a reduction in the pleasure obtained from other rewarding experiences, and an increase in negative affect. Their results also suggest that a smoking lapse could reestablish these affective and reward-related functions, resulting in a high probability of smoking relapse.

In fact, BA integrated in smoking cessation intervention has shown preliminary efficacy for depressive symptoms and improving smoking cessation outcomes among adult smokers with depression. MacPherson and colleagues [31] carried out a pilot study [*N* = 68] examining whether a behavioral activation treatment for smoking cessation (BATS) could improve abstinence rates and depressive symptoms compared to a standard smoking cessation treatment. They found that the BATS group had greater likelihood of point prevalence abstinence and lower depressive symptoms at the end of the treatment and at 6-month follow-up, compared with the standard smoking cessation treatment group. Also, a recent case series study [32] conducted with 3 inpatients (who participated in the open-label pilot phase of a larger ongoing RCT) at a substance use treatment center, found that the BA-Enhancing Smoking Cessation Program provides good smoking cessation results and important decreases in depressive symptoms.

Taken together, these previous findings suggest a potential benefit of adding BA components to a smoking cessation treatment. Through this intervention approach, exposure to positive reinforcing alternatives to smoking cigarettes would be increased, and the distress of the withdrawal syndrome could be reduced, resulting in a possible improvement of smoking abstinence rates and mood, even in smokers with lower depressive symptoms. Due to the novelty of this smoking cessation intervention, further research is needed with controlled trial designs, larger samples, and longer follow-up periods.

## Aims

The main aims of the present randomized controlled trial are: 1) to assess the efficacy (smoking abstinence rates) of a cognitive-behavioral smoking cessation treatment with elements from behavioral activation for managing depressed mood, compared with a standard smoking cessation treatment, at the end of treatment, and at 3-, 6-, and 12-month follow-ups; and 2) to assess whether the applied cognitive-behavioral smoking cessation treatment plus behavioral activation improves depressed mood, compared with a standard smoking cessation treatment, at the end of treatment and at 3-, 6-, and 12-month follow-ups.

## Method

### Study design

**Fig. 1:**
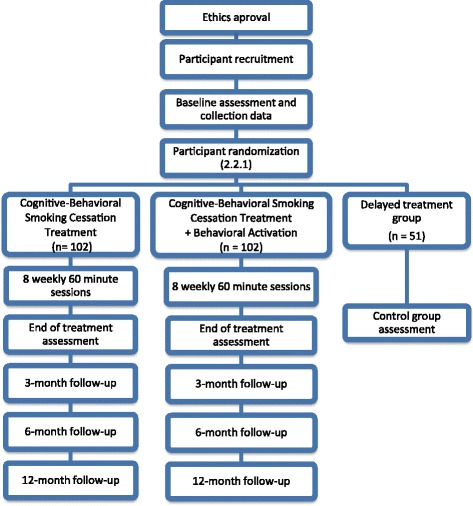
Consort Diagram of Trial Progression

A three-arm, single blind superiority randomized controlled design is proposed to assess the effectiveness of a cognitive-behavioral intervention for smoking cessation with components of BA. The overall study design is summarized in Fig. 1.

### Recruitment

Participants will be recruited through the media, posters in healthcare centers, word of mouth, or they will be referred to the unit by their primary care physician or other specialized services of the healthcare system. The different phases of the study (assessment, treatment, and follow-ups) will be carried out at the Smoking Cessation and Addictive Disorders Unit of the University of Santiago de Compostela, Galicia (Spain). Before participants enroll in the study, informed consent for participation will be obtained.

### Target population, blinding, randomization, and procedure

Sample selection will be carried out according to the following inclusion criteria: aged 18 or over; wishing to participate in the treatment program; providing written informed consent; and smoking 8 or more cigarettes per day. Exclusion criteria will be: a diagnosis of severe mental disorder (bipolar disorder and/or psychotic disorder); concurrent substance use disorder (alcohol, cannabis, stimulant, hallucinogen and/or opioid); having participated in the same or similar treatment over the previous year or having received pharmacological smoking cessation treatment (nicotine replacement therapy, bupropion, or varenicline) over the previous year; presence of a high life-risk pathology that would require immediate individual intervention (i.e., recent myocardial infarction); smoking tobacco products other than cigarettes (i.e., cigars); or failing to attend the first treatment session.

Two baseline assessment sessions will be carried out in a face-to-face interview. Researchers conducting such assessments will be blind to group allocation, which will occur subsequently. Due to the nature of the study, participants will be aware that they will be assigned to one of the three arms.

The randomization will be conducted according to a computer generated allocation sequence (ratio: 2.2.1.): (1) standard cognitive-behavioral smoking cessation treatment; (2) standard cognitive-behavioral smoking cessation treatment plus behavioral activation; or (3) a 3-month delayed treatment control group. After that, both active treatments will be administered in eight weekly 60-min sessions.

At the end of treatment, there will be a post-treatment assessment (during session 8) and face-to-face follow-ups at 3, 6, and 12 months (Fig. 1). Self-reported abstinence at the end of treatment and at the follow-ups will be corroborated by measures of CO in expired air. In those cases in which it is not possible to locate the participants, they will be considered to be smokers, and at the same level (in terms of tobacco-related variables) as at the baseline assessment.

### Sample size and power analysis

Previous studies of cognitive behavioral interventions for smoking cessation [20, 33] and studies of cognitive behavioral interventions for smoking cessation with components targeting depressive symptoms [24, 31] suggest that quit rates after treatment may range between 28% and 61.9%. The sample size for this study was calculated using G*Power3 Software [34]. To detect a 20% difference between the two active groups in the proportion of individuals with tobacco abstinence after treatment, with a power of 80% and a significant *p*-value of 0.05, a total of 102 participants per active group will be required. The sample size of the delayed-treatment control group will be 51 participants. We have chosen an unbalanced design because larger abstinence rates are expected in both active groups as compared to the delayed-treatment control group.

### Measures

Sociodemographic characteristics, smoking history, smoking cessation treatment history, depression history, and depression treatment history will be assessed using a face-to-face structured interview. In addition, the following instruments will be used (Table 1 lists the measures collected at each time point):

- Smoking Habit Questionnaire [35], consisting of 56 items designed to gather information both on sociodemographic variables (gender, age, marital status, educational level) and tobacco use (i.e., number of cigarettes smoked per day).
- Fagerström Test for Cigarette Dependence (FTCD) [36]. It is made up of six items for the assessment of cigarette dependence. Scores ≥6 are considered to be indicative of dependence [37]. Cronbach’s alpha coefficient was.60 in studies conducted in Spain [38].
- Nicotine Dependence Syndrome Scale (NDSS) [39, 40]. Questionnaire based on multidimensional conceptualization of substance dependence as a syndrome. The reliability of the general factor that evaluates nicotine dependence is good, Cronbach’s alpha was.80.
- Nicotine Withdrawal Scale Minnesota (MNWS) [41]. This is an 8-item scale measuring nicotine withdrawal symptoms (depression, insomnia, irritability/frustration/anger, anxiety, difficulty concentrating, restlessness, increased appetite/weight gain) and craving (desire or urge to smoke, which is considered independently). It is an instrument with good reliability, with a Cronbach’s alpha was.85.
- Structured Clinical Interview, according to DSM-5 criteria for assessing tobacco use disorder.
- Screening Questionnaire Major Depressive Episode (MDE) [42]. This is an instrument to detect past and current major depressive episodes.
- Beck Depression Inventory II (BDI-II) [43, 44]. This is a 21-item self-report scale measuring current depressive symptoms. The internal consistency obtained in Spanish sample with Cronbach’s alpha was.90.
- Hamilton Depression Rating Scale (HDRS) [45, 46]. This instrument was designed to quantitatively assess the severity of depressive symptoms and to monitor changes. The internal consistency obtained with a Cronbach’s alpha was.72.
- The Environmental Reward Observation Scale (EROS) [47, 48]. This is a brief self-report measure designed to obtain efficient, reliable, and valid information on the amount and availability of environmental reward. The Spanish version has a good internal consistency; Cronbach’s alpha was.86.
- Behavioral Activation for Depression Scale (BADS) [49, 50]. This 25-item questionnaire was designed to measure four basic dimensions of the behavioral activation model: Activation, Avoidance/Rumination, Work/School Impairment, and Social Impairment. The scale shows good internal consistency with Cronbach’s alpha was .90 for the total score, .81 for the Activation subscale, .82 for the Avoidance/Rumination subscale, .76 for the Work/School Impairment subscale, and .88 for the Social Impairment subscale.
- Ruminative Response Scale (RRS) [51, 52]. The RRS is a 22-item self-report for assessing ruminative coping responses to depressed mood. Treynor and colleagues [53] found that 12 items from the RRS overlapped with depressive symptoms, so that the resulting 10-item version will be used. The RRS Spanish version has been found to show adequate psychometric properties with Cronbach’s alpha scores for brooding and reflection of .73 and .72, respectively
- UCLA Loneliness Scale (Version 3) [54, 55]. This is a highly reliable instrument to measure loneliness, with a Cronbach’s alpha was .94.
- Carbon monoxide in expired air assessment. We will use the Micro + Smokerlyzer (Bedfont Scientific Ltd., Maidstone, Kent, U.K.) to measure carbon monoxide (CO) in expired air so as to corroborate self-reported abstinence at the end of treatment and at follow-ups, as suggested in previous studies [56].

**Table 1:** Timeline for data collection across the trial

Measures	Measurement time-point				
	Baseline	End of treatment	3-month follow-up	6-month follow-up	12-month follow-up
Smoking Habit Questionnaire	X				
TUS, Tobacco DSM-5 criteria	X				
MDE	X				
UCLA	X				
RRS	X				
FTCD	X	X	X	X	X
+NDSS	X	X	X	X	X
MNWS	X	X	X	X	X
BDI-II	X	X	X	X	X
HRDS	X	X	X	X	X
EROS	X	X	X	X	X
BADS	X	X	X	X	X
CO	X	X	X	X	X

*TUS* Tobacco Use Disorder, *MDE* Screening Questionnaire Major Depressive Episode; *UCLA* Loneliness Scale; *RRS* Ruminative Response Scale; *FTCD* Fagerström Test of Cigarette Dependence; *NDSS* Nicotine Dependence Syndrome Scale; *MNWS* Minnesota Nicotine Withdrawal Scale; *BDI-II* Beck Depression Inventory II; *HDRS* Hamilton Depression Rating Scale; *EROS* Environmental Reward Observation Scale; *BADS* Behavioral Activation for Depression Scale; *CO* carbon monoxide in expired air

### Intervention conditions

#### Standard cognitive-behavioral smoking cessation treatment

The standard cognitive-behavioral smoking cessation treatment is a manualized treatment for tobacco dependence, called “Smoking Cessation Program” [57]. The treatment components are: treatment contract, self-report and graphic representation of cigarette consumption, information about tobacco, nicotine fading [change of cigarette brands each week, progressively decreasing the intake of nicotine and tar), stimulus control, activities to prevent withdrawal syndrome, physiological feedback (CO in expired air) on cigarette consumption, and relapse-prevention strategies (assertion training, problem-solving training, changing tobacco-related misconceptions, management of anxiety and anger, exercise, weight control, and self-reinforcing). Treatment will be delivered in eight 60-min sessions over 8 consecutive weeks.

#### Standard cognitive-behavioral smoking cessation treatment and behavioral activation

Behavioral Activation will be applied along with the previously described standard cognitive-behavioral smoking cessation treatment. The treatment elements are the above-mentioned ones plus the following: analysis of the relationship between behavior and mood, identification of situations and behaviors that decrease mood, identifying avoidance behaviors, and identifying rumination and worry, self-report of pleasant daily activities, pleasant activity scheduling to increase engagement in rewarding activities and to reduce patterns of behavioral avoidance. Treatment will be delivered in eight 60-min sessions over 8 consecutive weeks.

#### Treatment delayed control group

It will be a delayed-treatment control group for a period of 3 months. After the 3-month period, another assessment will be carried out, and then participants will be offered to participate in a smoking cessation treatment (Table 2).

**Table 2:** Summary of session-by-session outline of intervention procedures

Ss	Cognitive-behavioral Smoking Cessation Treatment (CBSCT)	CBSCT + Behavioral Activation
1	Overview of treatment Smoking cessation treatment rationale Review self-monitoring (tracking cigarettes and smoking antecedents and consequences) Indications about how to graphically represent the No. of cigarettes/day Reasons for smoking and for quitting Discuss smoking history and quit experiences Explain and provide written materials about tobacco, nicotine dependence, smoking health consequences and quit smoking benefits Explain nicotine fading through brand change Physiological feedback (CO) As homework: - Brand change - Communicate to at least one person [family, friend, coworker, etc.) that he/she is trying to quit smoking in the next 30 days - Not smoke more cigarettes than the average of those smoked the previous week - Leave a third of the cigarette without smoking - Refuse cigarette offers	Overview of treatment Smoking cessation treatment rationale Review self-monitoring (tracking cigarettes and smoking antecedents and consequences) Indications about how to graphically represent the No. of cigarettes/day Reasons for smoking and for quitting Discuss smoking history and quit experiences Explain and provide written materials about tobacco, nicotine dependence, smoking health consequences and quit smoking benefits Explain nicotine fading through brand change Physiological feedback (CO) As homework: - Brand change - Communicate to at least one person (family, friend, coworker, etc.) that he/she is trying to quit smoking in the next 30 days - Not smoke more cigarettes than the average of those smoked the previous week - Leave a third of the cigarette without smoking - Refuse cigarette offers
2	Check homework and nicotine fading compliance Continue smoking self-monitoring and analyze smoking behavior during the week Physiological feedback (CO) Discuss brand change difficulties New brand change and reduce No. of cigarettes Review importance of social support Introduce stimulus control technique to remove situations conditioned to smoking Nicotine withdrawal and strategies to avoid it Breathing exercises and relaxation techniques (practice as homework)	Check homework and nicotine fading compliance Continue smoking self-monitoring and analyze smoking behavior during the week Physiological feedback (CO) Discuss brand change difficulties New brand change and reduce No. of cigarettes Review importance of social support Introduce stimulus control technique to remove situations conditioned to smoking Nicotine withdrawal and strategies to avoid it Breathing exercises and relaxation techniques (practice as homework)
3	Check nicotine fading, cigarette reduction and stimulus control compliance Check breathing exercises compliance and strategies to avoid withdrawal symptoms Physiological feedback (CO) Continue cigarette self-monitoring and analyze smoking behavior New brand change and reduce No. of cigarettes Continue stimulus control technique Explain and provide written materials for weight control and exercise Continue with breathing exercises and strategies to avoid withdrawal symptoms	Check nicotine fading, cigarette reduction and stimulus control compliance Check breathing exercises compliance and strategies to avoid withdrawal symptoms Physiological feedback (CO) Continue cigarette self-monitoring and analyze smoking behavior New brand change and reduce No. of cigarettes Continue stimulus control technique Explain and provide written materials for weight control and exercise Continue with breathing exercises compliance and strategies to avoid withdrawal symptoms Rationale of mood influence in smoking cessation [provide written materials) Homework: daily activities self-monitoring
4	Check nicotine fading, cigarette reduction and stimulus control compliance Check breathing exercises compliance and strategies to avoid withdrawal symptoms Physiological feedback (CO) Continue smoking self-monitoring and analyze smoking behavior New brand change and reduction of No. of cigarettes Continue stimulus control technique Stress and anxiety management strategies Problem-solving training	Check nicotine fading, cigarette reduction and stimulus control compliance Check breathing exercises compliance and strategies to avoid withdrawal symptoms Physiological feedback (CO) Continue smoking self-monitoring and analyze smoking behavior New brand change and reduction of No. of cigarettes Continue stimulus control technique Stress and anxiety management strategies Check activity scheduling and encourage recognizing patterns of depressed behavior and the way in which engaging in enjoyable and important activities may impact their overall mood Homework: continue activity scheduling, create a pleasant activities list and choose one to do during the week
5	Check nicotine fading, cigarette reduction, and stimulus control compliance Physiological feedback (CO) Continue smoking self-monitoring and analyze smoking behavior New brand change and reduction of No. of cigarettes Management of anxiety and anger Self-reinforcing Changing tobacco-related misconceptions	Check nicotine fading, cigarette reduction, and stimulus control compliance Check activity scheduling, pleasant activities list elaboration, and pleasant activity compliance Physiological feedback (CO) Continue smoking self-monitoring and analyze smoking behavior New brand change and reduction of No. of cigarettes Management of anxiety and anger Self-reinforcing Changing tobacco-related misconceptions Problem-solving training Recognize avoidance behavior and impact on mood Activity scheduling and engagement in 2 pleasant activities/week
6	Quitting experience and withdrawal symptoms Physiological feedback (CO) Discuss and plan for high-risk lapse and relapse situations Motivating factors for maintaining abstinence Benefits of quitting smoking Common barriers for maintaining abstinence	Quitting experience and withdrawal symptoms Physiological feedback (CO) Discuss and plan for high-risk lapse and relapse situations Motivating factors for maintaining abstinence Benefits of quitting smoking Common barriers for maintaining abstinence Ruminative thoughts, smoking cessation process, and relapse Check activity scheduling and pleasant activity compliance Activity scheduling for the next week and engagement in 2 pleasant activities/week
7	Quitting experience and withdrawal symptoms Physiological feedback (CO) Discuss and plan for high-risk lapse and relapse situations Motivating factors for maintaining abstinence Benefits of quitting smoking Strategies for relapse prevention	Quitting experience and withdrawal symptoms Physiological feedback (CO) Discuss and plan for high-risk lapse and relapse situations Motivating factors for maintaining abstinence Benefits of quitting smoking Review how behavioral activation impacts their overall mood Review avoidance behavior and ruminative thoughts’ significance Strategies for relapse prevention
8	Physiological feedback (CO) Managing the future as ex-smokers Encouragement for abstinence maintenance Support for lapses and relapse Review motivating factors, lifestyle changes, physical and cognitive-behavioral health improvement Treatment conclusion and management of potential obstacles	Physiological feedback (CO) Managing the future as ex-smokers Encouragement for abstinence maintenance Support for lapses and relapse Review motivating factors, lifestyle changes, physical and cognitive-behavioral health improvement Review BA strategies Treatment conclusion and management of potential obstacles

### Therapist

Trained therapists (Master level in clinical or counseling psychology) will conduct the assessment and intervention sessions.

### Treatment manual

Both treatments have been manualized with the aim of training the therapist and improving the intervention implementation. Both of them include a detailed session-by-session protocol and follow-up procedures.

### Intervention fidelity

All sessions will be video-recorded to supervise the therapist and to assess intervention fidelity. Study supervisors will assess fidelity through the random visualization of treatment sessions in both active arms.

### Outcomes

#### Primary outcome measures

- Point-prevalence abstinence defined according to Russell Standard (RS) criteria [56]. Participants will be considered abstinent if they report abstinence, not even a puff of a cigarette, for ≥24 h at the end of treatment, and for ≥7 days prior to follow-up day at the 3-month follow-up, and have an expired carbon monoxide reading of ≤10 ppm. At the 6- and 12-month follow-ups, participants will be considered abstinent if they report abstinence, not even a puff of a cigarette, for ≥30 days prior to follow-up day, and have an expired carbon monoxide reading of ≤10 ppm.
- Significant change in depressive symptoms on the Beck Depression Inventory-II (BDI-II) from baseline to the end of treatment, and at 3-, 6-, and 12-month follow-up.
- Significant change in depressive symptoms on the Hamilton Depression Rating Scale (HDRS) from baseline to the end of treatment, and at 3-, 6-, and 12-month follow-up.

### Secondary outcome measures

- Continuous abstinence: in accordance with the Russell Standard [56], we will consider that participants have achieved continuous abstinence if they report not having smoked more than five cigarettes from the start of the abstinence period, and have an expired carbon monoxide reading of ≤10 ppm.
- Reduction of cigarette consumption by 50% or more between baseline and each follow-up will be calculated by the mean number of cigarettes smoked at the end of treatment and in the 7 days prior to the 3-month follow-up. At 6-, and 12-month follow-ups, it will be calculated by the mean number of cigarettes smoked in the past 30 days.
- Significant change in the Environmental Reward Observation Scale (EROS) scores from baseline to the end of treatment, and at 3-, 6-, and 12-month follow-up.
- Significant change in the Behavioral Activation for Depression Scale (BADS) scores from baseline to the end of treatment, and at 3-, 6-, and 12-month follow-up.

### Data management and confidentiality

Data will be collected from participants both in paper and electronic format. To make anonymization possible, a unique identity code number for use on trial documents and electronic database will be assigned to each participant. Data collected on paper forms will be kept in a locked filing cabinet. Electronic data will be kept in password-protected computer folders at the Smoking Cessation and Addictive Disorders Unit (University of Santiago de Compostela). Only authorized trial staff will have access to trial documentation.

### Ethical principles

The Bioethics Committee of the University of Santiago de Compostela approved this study, which is registered with the international standard randomized controlled trial number NCT02844595 (www.clinicaltrials.gov). The participants will be provided with both oral and written information regarding the study prior to obtaining their informed consent.

### Statistical analysis

All data analysis will follow the intention-to-treat principle. This conservative approach aims to minimize selection bias through the inclusion in the primary analysis of data from all the participants randomized to each group, including those who drop out of the study.

We will conduct descriptive analysis to summarize the characteristics of the total sample, and the characteristics of the participants in each of the three groups. The main analysis will be a comparison between both active groups and the control group of the proportions of abstinent smokers at the end of treatment, and between BA intervention group and standard intervention group at the end of treatment, and at 3-, 6-, and 12-month follow-ups, through chi-square tests and odds ratios with 95% confidence intervals. A *t*-test analyses will be conducted to determine whether there were any significant differences between the two active treatment groups and the control group in continuous variables such as BDI-II score. We also will conduct mediation analysis, multiple regression analysis, repeated measures ANOVA, and survival analysis.

The study results will be reported in accordance with the Consolidated Standards of Reporting Trials (CONSORT) 2010 statements [58, 59] and Standard Protocol Item: Recommendations for Interventional Trials (SPIRIT) guidelines [60, 61].

## Discussion

To our knowledge, this is the first randomized controlled trial conducted evaluating the efficacy of a cognitive-behavioral smoking cessation intervention (CBSCI) with components of Behavioral Activation (BA). As the relationship between depressive symptoms, even at subclinical levels, and difficulties in abstinence achievement and maintenance is well known, we expect that this intervention will allow obtaining higher abstinence rates, lower relapse rates, and mood improvement after the treatment and at long-term.

This study has significant strengths: (1) to date, it will be the largest trial to address the clinical-effectiveness of BA integrated into a cognitive-behavioral smoking cessation treatment; (2) follow-up assessments will be carried out at 3-, 6- and 12-month post-treatment in both active groups (CBSCI vs. CBSCI + BA), which will provide an opportunity to evaluate the long-term treatment effects on abstinence rates and mood; and (3) participants will be included regardless of current depressive symptom level, which will allow us to investigate the influence of subclinical depressive symptomatology in treatment outcomes.

We expect to find that an effective, brief, and flexible intervention for depression such as BA could be implemented as a part of a cognitive-behavioral treatment for smoking cessation in order to improve its efficacy. The findings obtained will have significant clinical implications. Because of the relevance of depressive symptoms in the smoking cessation process, this intervention could be a good option to address two problems that have a very high impact on people’s health and quality of life.

## Trial status

The study started in January 2016, and the recruitment is ongoing.
